# Small Strain Stiffness of Unsaturated Sands Containing a Polyacrylamide Solution

**DOI:** 10.3390/ma10040401

**Published:** 2017-04-11

**Authors:** Jongwon Jung, Taeseo Ku, Jaehun Ahn

**Affiliations:** 1 School of Civil Engineering, Chungbuk National University, Cheongju, Chungbuk 28644, Korea; jjung@chungbuk.ac.kr; 2 Department of Civil and Environmental Engineering, National University of Singapore, Singapore 117576, Singapore; ceekt@nus.edu.sg; 3 Department of Civil and Environmental Engineering, Pusan National University, Busan 46241, Korea

**Keywords:** small strain stiffness, shear wave velocity, polyacrylamide, unsaturated sand

## Abstract

Sand improvements using organic agents have shown promising results. Polyacrylamide is one possible organic agent, which has been shown to influence the shear strength, stiffness, soil remediation, and erosion resistance of geomaterials. In this study, we explored the shear wave velocity (S-wave) and water retention curves of unsaturated sands containing polyacrylamide solutions. The shear wave velocity was measured during the water retention curve measurement tests according to the variation of the degree of saturation. The experimental setup was verified through comparison of the measured water retention curves with the published data. The results show that (1) the S-wave velocity of saturated sands increases with polyacrylamide concentration; (2) as the degree of saturation decreases, the S-wave velocity increases; (3) near the residual water (or polyacrylamide solution) saturation, the S-wave velocity increases dramatically; (4) as the degree of saturation decreases, the S-wave velocity at unsaturated conditions increases with any given water (or polyacrylamide solution) saturation, like the water retention curves; (5) the S-wave velocity increases with the increase in capillary pressure; and (6) the predicted S-wave velocity at a given degree of saturation is slightly overestimated, and the modification of the equation is required.

## 1. Introduction

Soil improvements using organic agents such as polyacrylamide, xanthan gum, and surfactants have been developed which have shown promising results at improving the shear strength, stiffness, soil remediation, and erosion resistance of geomaterials [1,2,3,4,5,6,7,8,9]. Amongst them, polyacrylamide (PAM) influences irrigation through increased water infiltration and decreased erosion by its capacity to absorb and store water [10,11,12,13]. Additionally, polyacrylamide (PAM) has been shown to increase structural stability [14].

Most soils are under unsaturated conditions in nature. In unsaturated soils, the capillary pressure (*P_c_*) is determined by the difference between the air- and water-pressures, which is affected by water saturation (*S_w_*) in soils. Equation (1) shows the most popular theoretical equation [15] to represent the relation between water saturation (*S_w_*) and capillary pressure (*P_c_*) in unsaturated soils.

(1) $$P_{c}=P_{o}{\left[{\left(\frac{S_{w}-S_{r}}{1-S_{r}}\right)}^{-\frac{1}{m}}-1\right]}^{1-m}$$

where *P_c_* is the capillary pressure, *P_o_* is the capillary air entry pressure, *S_w_* is the water saturation, *S_r_* is the residual water saturation, and *m* is the fitting parameter.

The relation between water saturation (*S_w_*) and capillary pressure (*P_c_*) in unsatureated soils influences the relative permeability of fluids [16,17,18,19,20,21,22], water storage capacity of the soils [23], shear strength [24,25,26], as well as the stiffness and volume change [27,28,29,30,31].

However, the stiffness changes of unsaturated sands containing polyacrylamide (PAM) solution have not yet been understood. Thus, in this study we investigate the water (or polyacrylamide solution) saturation (*S_w_*) and capillary pressure (*P_c_*) relations as well as explore the stiffness of unsaturated sands through shear wave velocity measurements.

## 2. Experimental Study

### 2.1. Materials

**Figure 1:**
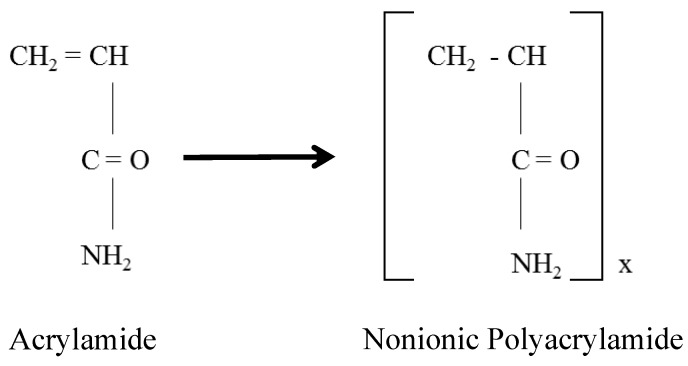
Chemical structure of polyacrylamide.

Commercial polyacrylamide (Acros Organics) was used in this study. The polyacrylamide is a linear polymer produced by acrylamide, whose chemical structure is (C_3_H_5_NO)*_n_* (Figure 1). Ottawa sand F-75 mixed with a variety of polyacrylamide concentrations (i.e., 0, 2, 5, and 7.5 g/L) were prepared. More than 95% of the sands were within the range of 0.2 mm–0.5 mm (particle size). The effective size of the sand, *D*_10_, was 0.14 mm, and its coefficients of uniformity (*C_u_*) and curvature (*C_c_*) were 1.46 and 1.01, respectively.

### 2.2. Experimental Procedure

**Figure 2:**
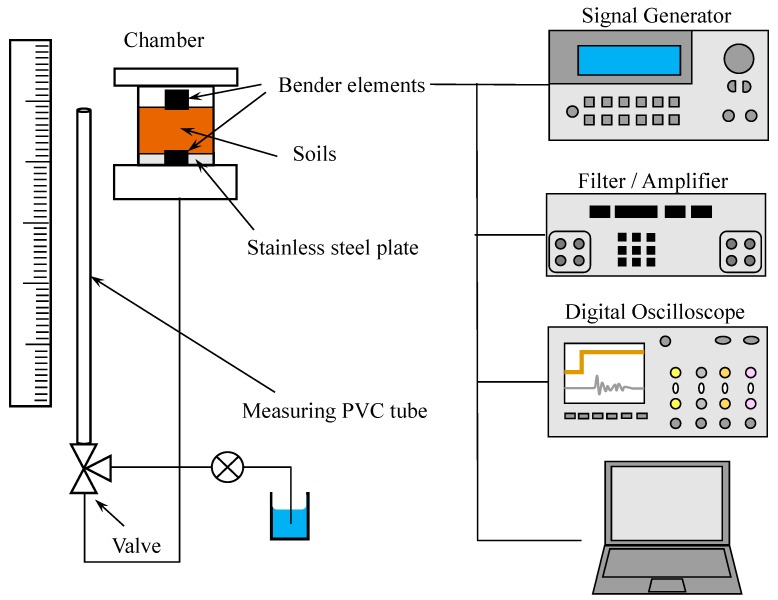
Experimental setup for water retention curve and S-wave velocity measurement tests.

Figure 2 shows the experimental setup for the water retention curve (or soil-water characteristic curve) tests including the bender elements to measure the shear wave velocity. One stainless steel plate with 50 kPa air-entry pressure (diameter = 8.03 cm and thickness = 2.0 cm) was placed on the bottom of the chamber (inner diameter, ID = 7.62 cm and height = 8.92 cm). The polyacrylamide saturated sands were packed in the chamber (volume of sand = 251.7 cm^3^, dry bulk density = 1.64 g/cm^3^) with constant porosity *n* = 0.381 kept for all tests. For the perfect saturation, the wetting method was used. One fifth of the chamber was filled with the polyacrylamide solution first and soils were then placed into the polyacrylamide solution, which was repeated until the chamber was filled with sand. Another stainless steel plate was placed on the top of the sand. Two bender elements were attached to stainless steel plates that were located on the top and bottom of the sands, which acted as a source and a receiver, respectively. The shear wave generated by the source bender element connected to a function generator (33210A, Agilient, Santa Clara, CA, USA) (square wave of frequency = 20 Hz and amplitude = 10 V) traveled through the soils, and arrived at the other bender element that acted as the signal receiver. The receiver bender element was connected to the filter amplifier (3364, Krohn-Hite, Brockton, MA, USA), which in turn was connected to the digital oscilloscope (DSO6014A, Agilent, Santa Clara, CA, USA). A total of 2048 signals were stacked to reduce the influence of uncorrelated noise. The travel time of the shear wave was determined using the digitized signal as recorded by the oscilloscope, while the tip-to-tip distance (the distance from the tip of the source bender element to the tip of the receiver bender element) was used as the travel distance [32].

The hanging water column method [33,34,35] was used to simulate the capillary pressure (*P_c_*)-water saturation (*S_w_*) relation. The bottom portion of the sand chamber was connected to a vertical PVC tube (ID = 8.0 mm). The capillary pressure (*P_c_*) was controlled by the elevation change of the air-water interface in the PVC tube. For each capillary pressure, the shear wave velocity was measured. During the shear wave velocity measurements, the valve located between the sand chamber and the vertical PVC tube was closed to avoid vibration effects by the bender element on the capillary pressure (*P_c_*)-water saturation (*S_w_*) relation. The top portion of the PVC tube and the soil chamber were simply vented to the room air to maintain the atmospheric pressure condition [34].

## 3. Results and Discussion

**Figure 3:**
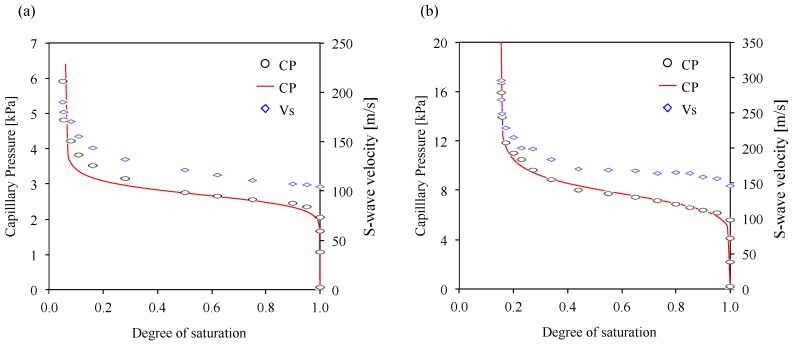

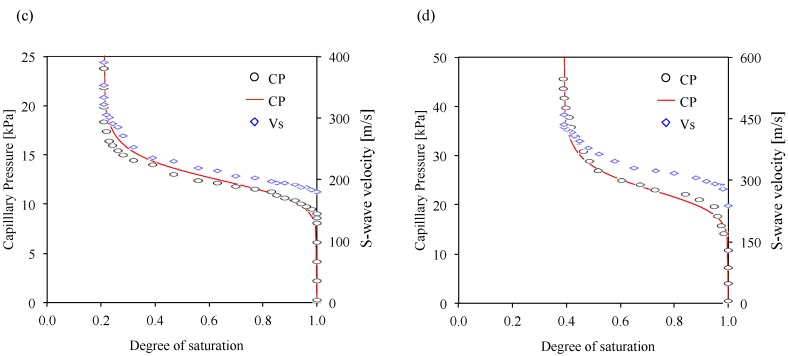
Water retention curve and S-wave velocity results. The black points represent the capillary pressure-degree of saturation in this study. The blue points present S-wave velocity changes according to the variation of the degree of saturation. The continuous line represents the results from the van-Genuchten model. (**a**) De-ionized water; (**b**) 2.5 g/L polyacrylamide solution; (**c**) 5.0 g/L polyacrylamide solution and (**d**) 7.5 g/L polyacrylamide solution.

Figure 3 shows the water retention curves (or soil-water characteristic curve) and shear wave (S-wave) velocity at different polyacrylamide concentrations.

### 3.1. Water Retention Curves

The four water retention curves show a good consistency with the van Genuchten models (Equation (1)). The *m*-values for the van Genuchten models have a range of 0.93–0.94, which are obtained from a previous study [36]. The values measured in this study are shown as data points, and then the continuous curves are fitted using the van Genuchten models (Equation (1)) in Figure 3. The results show three clear zones; the capillary saturation, desaturation, and residual saturation zones, similar to previous studies [37,38,39]. As the polyacrylamide concentration increases, (1) the capillary air-entry pressure increases; (2) the residual water saturation values in the air-water system are about 0.05, which is consistent with the previous studies [40,41]; (3) the residual water (or polyacrylamide solution) saturation increases due to the higher bonding strength between the polyacrylamide solution and the silica sand surface [7,9] that also causes the strength of the sand containing the polyacrylamide solution to increase [6,8]; and (4) the water retention curves shift to higher capillary pressure with any given water saturation.

### 3.2. Shear Wave Velocity

**Figure 4:**
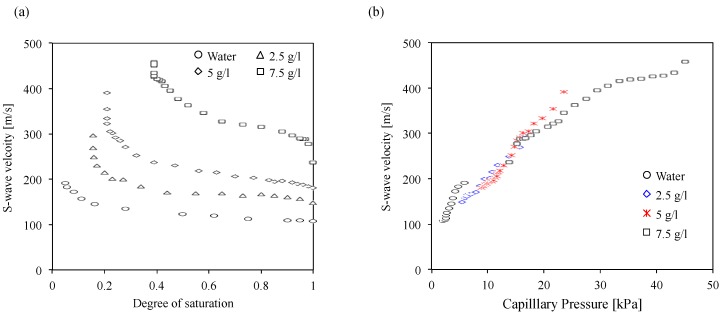
Degree of saturation and capillary pressure effects on the S-wave velocity. (**a**) S-wave velocity variation of unsaturated soils at different polyacrylamide concentrations; (**b**) S-wave velocity changes according to the variation of capillary pressure.

The measured shear wave (S-wave) velocities are plotted with the water retention curves in Figure 3, which shows that the general trends of the S-wave velocity are quite similar to the water rentention curves. The S-wave velocity increases with the decrease in the degree of saturation, which is consistent with the previous studies [42,43]. Additionally, the results present no change of the S-wave velocity in the capillary saturation zone, a small increase in the S-wave velocity in desaturation zone, and a dramatic increase in the S-wave velocity in the residual saturation zone with increased capillary pressure. Figure 4a compares the S-wave velocity change according to the variation of the degree of saturation at different polyacrylamide concentrations. The results show that (1) the S-wave velocity of the water-saturated soils is 110 m/s, which is consistent with a previous study [42]; (2) the S-wave velocity of saturated soils increases with increasing polyacrylamide concentration; (3) as the degree of saturation decreases, the S-wave velocity increases; (4) near the residual water (or polyacrylamide solution) saturation, the S-wave velocity increases dramatically; and (5) as the degree of saturation decreases, the S-wave velocity at unsaturated conditions increases with any given water (or polyacrylamide solution) saturation, like the water retention curves.

### 3.3. Capillary Pressure Effects

**Figure 5:**
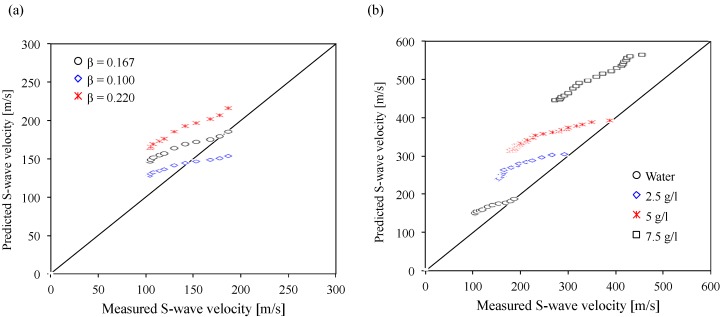
Comparison of the predicted S-wave velocity with the measured S-wave velocity. (**a**) *β*-value effects on the predicted S-wave velocity; (**b**) Comparison of the predicted S-wave velocity with the measured S-wave velocity in all polyacrylamide concentrations.

Figure 4b shows the S-wave velocity variation according to capillary pressure changes. The results show that the S-wave velocity increases with increased capillary pressure. The S-wave velocity is determined by the mass density of the soil mass and the stiffness of the granular skeleton [44,45], which is affected by the degree of saturation. The capillary pressure increases with decreased water saturation, which causes the increase of the stiffness of the granular skeleton [42]. Thus, the S-wave velocity increases in line with increases in capillary pressure, as shown in Figure 4b. The S-wave velocity at a given degree of saturation can be predicted by the S-wave velocity at the saturated condition (*S* = 1) considering the change in mass density of the soils using Equation (2) [42].

(2) $${\left[V_{s}\right]}_{s}={\left[V_{s}\right]}_{s=1\;}{\left[1+\frac{2\sigma_{eq}^{\prime }}{\left(1+K_{o}\right)\sigma_{v}^{\prime }}\right]}^{\beta }\sqrt{\frac{e+G_{s}}{eS+G_{s}}}$$

where, $\sigma_{eq}^{\prime }$ is the equivalent stress due to the capillary pressure at a given degree of saturation, $\sigma_{v}^{\prime }$ is the effective vertical stress, *e* is the void ratio, *Gs* is the specific gravity, *K_o_* is the coefficient of lateral earth pressure at rest, *S* is the degree of saturation, [*V_s_*]*_s_* is the S-wave velocity at a given degree of saturation, [*V_s_*]*_s=_*_1_ is the S-wave velocity at the saturated condition, and *β* is an experimentally determined parameter. *β* is 1/6 for the glass beads and 0.22 for the sandboil sand that is a natural soil from a paleoliquefaction site in mid-America [42]. The predicted S-wave velocity at a given degree of water saturation is estimated using Equation (2) within the range of *β =* 0.10–0.22, and is compared with the measured S-wave velocity in this study (Figure 5a). The results show that the predicted S-wave velocity is mostly similar to the measured S-wave velocity when the *β*-value is 0.167 (Figure 5a), which is reasonable considering the *β-*value for the glass beads in a previous study (*β* = 1/6) [42].

Figure 5b presents the comparison of the predicted S-wave velocity in all polyacrylamide concentrations with the measured S-wave velocity when the *β*-value is 0.167. The results show that the predicted S-wave velocity values of sands containing polyacrylamide solutions are slightly higher than the measured values (Figure 5b). It implies that the maximum shear modulus (*G*_max_) of sands containing polyacrylamide solutions using the predicted S-wave velocity can be overestimated. Higher polyacrylamide concentrations cause the water (or polyacrylamide solution) retention curves to shift to higher capillary pressure at a given degree of saturation due to the bonding strength between the polyacrylamide solutions and the silica soil surfaces, and the higher viscosity of the polyacrylamide solutions [4,7,9]. However, the bonding strength by polyacrylamide solutions can have a slight influence on the increase in the S-wave velocity due to the lower shear stiffness of the polyacrylamide solutions as fluids. Thus, Equation (2) should be valid for calculating the S-wave velocity when the fluid in unsaturated soils is only water. The modified equation should be required for other fluids such as polyacrylamide solutions.

## 4. Conclusions

The water retention curves and S-wave velocity were measured in a homogeneous silica sand pack using an air-water (or polyacrylamide solutions) system with different polyacrylamide concentrations. Generally, the water retention curves showed that (1) the capillary air entry values increase with polyacrylamide concentration; (2) the water retention curves shifts to higher capillary pressure at a given water saturation; and (3) the residual saturation increases with polyacrylamide concentration. The water retention curves in this study were compared with the theoretical van Genuchten model using fitting parameters (i.e., *m*-values) obtained from a previous study, which showed a good consistency with each other. It implies that the experimental setup in this study is valid for simulating the water saturation-capillary relation, and for measuring the S-wave velocity.

Additionally, the S-wave velocity was measured according to the variation of the degree of saturation during the water retention curve measurement tests. The results showed that (1) the S-wave velocity of saturated sands increases with polyacrylamide concentration; (2) as the degree of saturation decreases, the S-wave velocity increases; (3) near the residual water (or polyacrylamide solution) saturation, the S-wave velocity increases dramatically; (4) the S-wave velocity at unsaturated conditions increases with any given water (or polyacrylamide solution) saturation like the water retention curves; (5) the S-wave velocity increases with the increase in capillary pressure; and (6) the predicted S-wave velocity at a given degree of saturation is slightly overestimated, and a new modified equation needs to be proposed in future studies.

## References

[1] Briscoe W.H., Klein J. (2007). Friction and adhesion hysteresis between surfactant monolayers in water. J. Adhes..

[2] Yoshizawa H., Chen Y.L., Israelachvili J. (1993). Fundamental mechanisms of interfacial friction. 1. Relation between adhesion and friction. J. Phys. Chem..

[3] Bate B., Zhao Q., Burns S. (2013). Impact of organic coatings on frictional strength of organically modified clay. J. Geotech. Geoenviron. Eng..

[4] Jung J., Jang J., Ahn J. (2016). Characterization of a polyacrylamide solution used for remediation of petroleum contaminated soils. Materials.

[5] Kang J., Sowers T.D., Duckworth O.W., Amoozegar A., Heitman J.L., Mclaughlin R.A. (2013). Turbidimetric determination of anionic polyacrylamide in low carbon soil extracts. J. Environ. Qual..

[6] Martin G., Yen T., Karimi S. Application of biopolymer technology in silty soil matrices to form impervious barriers. Proceeding of the Conference on 7th Australia New Zealand Conference on Geomechanics: Geomecahnics in a Changing Wolrd.

[7] Kavazanjian E., Iglesias E., Karatas I., Hamza M., Shahien M., El-Mossallamy Y. Biopolymer soil stabilization for wind erosion control. Proceeding of the Conference on the 17th International Conference on Soil Mechanics and Geotechnical Engineering.

[8] Cabalar A.F., Canakci H. Ground improvement by bacteria. Proceeding of the Conference on the 3rd Biot Conference on Poromechanics.

[9] Nugent R., Zhang G., Gambrell R. (2009). Effect of exopolymers on the liquid limit of clays and its engineering implications. J. Transp. Res. Board.

[10] Sojka R.E., Bjorneberg D.L., Entry J.A., Lentz R.D., Orts W.J. (2007). Polyacrylamide in agriculture and environmental land management. Adv. Agron..

[11] Inbar A., Ben-Hur M., Sternberg M., Lado M. (2015). Using polyacrylamide to mitigate post-fire soil erosion. Geoderma.

[12] Lentz R.D. (2015). Polyacrylamide and biopolymer effects on flocculation, aggregate stability, and water seepage in a silt loam. Geoderma.

[13] Lee S.S., Shah H.S., Awad Y.M., Kumar S., Ok Y.S. (2015). Synergy effects of biochar and polyacrylamide on plants growth and soil erosion control. Environ. Earth Sci..

[14] Mamedov A., Wagner L., Huang C., Norton L., Levy G. (2010). Polyacrylamide effects on aggregate and structure stability of soils with different clay mineralogy. Soil Sci. Soc. Am. J..

[15] Van Genuchten M.T. (1980). A closed-form equation for predicting the hydraulic conductivity of unsaturated soils. Soil Sci. Soc. Am. J..

[16] Assouline S. (2001). A model for soil relative hydraulic conductivity based on the water retention characteristic curve. Water Resour. Res..

[17] Campbell G.S. (1974). A simple method for determining unsaturated conductivity from moisture retention data. Soil Sci..

[18] Fischer U., Celia M.A. (1999). Prediction of relative and absolute permeabilities for gas and water from soil water retention curves using a pore-scale network model. Water Resour. Res..

[19] Mualem Y. (1986). Hydraulic conductivity of unsaturated soils: Prediction and formulas. Agronomy.

[20] Vogel T., Cislerova M. (1988). On the reliability of unsaturated hydraulic conductivity calculated from the moisture retention curve. Transp. Porous Media.

[21] Zhao X. (2014). Measurements and Transient Multistep Outflow Simulation of Soil-Water Characteristic Curve for Soils Modified with Biopolymers. Master’s Thesis.

[22] Javadi S., Ghavami M., Zhao Q., Bate B. (2016). Advection and retardation of non-polar contaminants in compacted clay barrier material with organoclay amendment. Appl. Clay Sci..

[23] Brady N.C., Weil R.R. (2010). Elements of the Nature and Properties of Soils.

[24] Fredlund D.G., Xing A., Fredlund M.D., Barbour S.L. (1996). The relationship of the unsaturated soil shear to the soil-water characteristic curve. Can. Geotech. J..

[25] Öberg A.L., Sällfors G. (1997). Determination of shear strength parameters of unsaturated silts and sands based on the water retention curve. Can. Geotech. J..

[26] Vanapalli S.K., Fredlund D.G., Pufahl D.E., Clifton A.W. (1996). Model for the prediction of shear strength with respect to soil suction. Can. Geotech. J..

[27] Delage P., Howat M., Cui Y. (1998). The relationship between suction and swelling properties in a heavily compacted unsaturated clay. Eng. Geol..

[28] Gens A., Alonso E.E. (1992). A framework for the behaviour of unsaturated expansive clays. Can. Geotech. J..

[29] Pedarla A., Puppala A.J., Hoyos L.R., Vanapalli S.K., Zapata C. (2012). Unsaturated soils: Research and Applications.

[30] Kang X., Bate B. (2016). Shear wave velocity and its anisotropy of polymer modified high volume class F fly ash-kaolinite mixtures. J. Geotech. Geoenviron. Eng..

[31] Kang X., Kang G.-C., Bate B. (2014). Shear wave velocity anisotropy of kaolinite using a floating wall consolidometer-type bender element testing system. Geotech Test. J..

[32] Lee J.S., Santamarina J.C. (2005). Bender elements: Performance and signal interpretation. J. Geotech. Geoenviron. Eng..

[33] Haines W.B. (1930). Studies in the physical properties of soil. V. The hysteresis effect in capillary properties, and the modes of moisture distribution associated therewith. J. Agric. Sci..

[34] Tokunaga T.K., Wan J., Olson K.R. (2002). Saturation-matric potential relations in gravel. Water Resour. Res..

[35] ASTM Standard D 6836 (2003). Test Methods for Determination of the Soil Water Characteristic Curve for Desorption using a Hanging Column, Pressure Extractor, Chilled Mirror Hygrometer, And/Or Centrifuge.

[36] Jung J., Jang J. (2016). Soil-water characteristic curve of sediments containing a polyacrylamide solution. Geotech. Lett..

[37] Sillers W.S., Fredlund D.G., Zakerzadeh N. (2001). Mathematical attributes of some soil—water characteristic curve models. Geotech. Geol. Eng..

[38] Yang H., Rahardjo H., Leong E.C., Fredlund D.G. (2004). Factors affecting drying and wetting soil-water characteristic curves of sandy soils. Can. Geotech. J..

[39] Zhou J., Yu J.L. (2005). Influences affecting the soil-water characteristic curve. J. Zhejiang Univ. Sci..

[40] Gittins P., Iglauer S., Pentland C.H., Al-Mansoori S., Al-Sayari S., Bijeljic B., Blunt M.J. (2010). Nonwetting phase residual saturation in sand packs. J. Porous Media.

[41] Schroth M., Istok J., Ahearn S., Selker J. (1996). Characterization of Miller-similar silica sands for laboratory hydrologic studies. Soil Sci. Soc. Am. J..

[42] Cho G.C., Santamarina J.C. (2001). Unsaturated particulate materials—Particle-Level Studies. J. Geotech. Geoenviron. Eng..

[43] Alramahi B., Alshibli K.A., Fratta D., Trautwein S. (2008). A suction-control apparatus for the measurement of P and S wave velocity in soil. Geotech. Test. J..

[44] Roesler S.K. (1978). Anisotropic shear modulus due to stress anisotropy. J. Geochem. Eng..

[45] Santamarina J.C., Klein K., Fam M. (2001). Soils and waves: Particulate materials behavior, characterization and process monitoring. J. Soils Sediments.

